# New Hemodynamic Parameters in Peri-Operative and Critical Care—Challenges in Translation

**DOI:** 10.3390/s23042226

**Published:** 2023-02-16

**Authors:** Laura Bogatu, Simona Turco, Massimo Mischi, Lars Schmitt, Pierre Woerlee, Rick Bezemer, Arthur R. Bouwman, Erik H. H. M. Korsten, Jens Muehlsteff

**Affiliations:** 1 Biomedical Diagnostics Lab (BM/d), Eindhoven University of Technology, 5612 AZ Eindhoven, The Netherlands; 2 Patient Care and Measurements, Philips Research, 5656 AE Eindhoven, The Netherlands; 3 Department of Anesthesiology, Intensive Care and Pain Medicine, Catharina Ziekenhuis, 5623 EJ Eindhoven, The Netherlands

**Keywords:** hemodynamic monitoring, hemodynamic parameter measurements, patient monitoring, minimally invasive monitoring

## Abstract

Hemodynamic monitoring technologies are evolving continuously—a large number of bedside monitoring options are becoming available in the clinic. Methods such as echocardiography, electrical bioimpedance, and calibrated/uncalibrated analysis of pulse contours are becoming increasingly common. This is leading to a decline in the use of highly invasive monitoring and allowing for safer, more accurate, and continuous measurements. The new devices mainly aim to monitor the well-known hemodynamic variables (e.g., novel pulse contour, bioreactance methods are aimed at measuring widely-used variables such as blood pressure, cardiac output). Even though hemodynamic monitoring is now safer and more accurate, a number of issues remain due to the limited amount of information available for diagnosis and treatment. Extensive work is being carried out in order to allow for more hemodynamic parameters to be measured in the clinic. In this review, we identify and discuss the main sensing strategies aimed at obtaining a more complete picture of the hemodynamic status of a patient, namely: (i) measurement of the circulatory system response to a defined stimulus; (ii) measurement of the microcirculation; (iii) technologies for assessing dynamic vascular mechanisms; and (iv) machine learning methods. By analyzing these four main research strategies, we aim to convey the key aspects, challenges, and clinical value of measuring novel hemodynamic parameters in critical care.

## 1. Introduction

In peri-operative and critical care, assessment of hemodynamic function plays an essential role in the treatment of patients at risk of deterioration [1]. The principle of hemodynamic monitoring consists of interpreting a number of observations or measurements with reference to a model that encompasses the overall current understanding of cardiovascular physiology. For example, information on parameters such as cardiac output (CO), blood pressure (BP), and central venous pressure (CVP) is usually interpreted with respect to the Guytonian view of the circulation [2]. This enables a general assessment of cardiac function and fluid responsiveness based on a limited number of physiological measurements, supporting clinicians in detecting patient deterioration and monitoring therapy effectiveness [3].

In practice, the way in which measurements are performed for the purpose of hemodynamic assessment can differ greatly in terms of the clinical context: pathophysiology and progression of disease, the type of procedure and level of acuity, and further patient-specific requirements and restrictions. These factors determine the required level of measurement accuracy and frequency for adequate clinical decision support, which in turn determine the level of monitoring invasiveness, measurement location, and degree of automation [1]. Invasive methods involving arterial, venous, and pulmonary artery catheters enable accurate, continuous measurement of hemodynamic parameters. However, their clinical benefits are in many cases outweighed by the probability of complications associated with invasive lines, the dependence on resources and trained staff, and the uncertainty of whether invasive monitoring would actually lead to better patient outcomes when compared with non-invasive, intermittent, or less accurate monitoring [4].

To tackle the compromise between the reliability of measurements and their invasiveness, various low-risk hemodynamic monitoring technologies are being developed [5,6]. A large number of bedside monitoring options are becoming available in the clinic [7], including echocardiography, technologies involving electrical bioimpedance and bioreactance, and techniques based on calibrated and uncalibrated arterial pulse contour analysis [6]. Such methods are allowing for more continuous, accurate, minimally invasive hemodynamic management; this has led to a decline in the use of highly invasive monitoring such as pulmonary artery catheterization.

The new devices mainly aim to monitor the well-known hemodynamic variables that have been previously validated and extensively used in clinical practice. For example, novel pulse contour or bioreactance methods aim to measure widely used macrocirculatory variables such as central arterial BP, CO, and related indices—preload, afterload, and contractility. As a result, even though monitoring technologies are continuously evolving, the parameters most frequently used in standard practice remain largely unchanged. This applies especially to general wards, where it is difficult to reliably measure additional hemodynamic parameters beyond arterial BP, peripheral arterial oxygen saturation (SpO2), and cardiac rhythm via electrocardiogram (ECG) [8].

While hemodynamic monitoring has developed greatly over the last decade, a number of issues still remain due to the limited amount of information available for diagnosis and treatment.

For example, there is still uncertainty in the interpretation of widely used variables such as BP [9,10,11]. Even in the case of accurate, continuous BP measurements, the BP value is a late indicator of deterioration, and controversy exists in the clinical community regarding what is considered a “healthy” BP range. While recent findings suggest that a 65-mmHg mean arterial pressure (MAP) threshold should be regarded as an absolute minimum to prevent harmful hypotensive conditions, this contradicts the movement towards personalized BP thresholds in fluid/vasopressor therapy protocols [9,12]. This leaves the use of BP as a therapeutic target open for discussion. One aspect of this discussion is that recent findings related to the microcirulatory function in critically ill patients have shown how micro- and macrocirculation can sometimes be dissociated, especially in cases of sepsis [13], and that tissue hypoperfusion may exist under conditions of normal and high BP. Monitoring the microcirculation, however, is a cumbersome task to date and not part of standard clinical guidelines or protocols. Another recognized need is the earlier detection of deterioration in low-acuity settings, the wards requiring the most immediate improvement [14]. Missed patient deterioration as a result of insufficient ward monitoring is considered one of the main factors in the 4.2 million cases worldwide per year in which patients die within 30 days after surgery. At the same time, however, the existing ward measurements lead to frequent alarms that are unspecific and have a false alarm rate of 90% [15]. This further presses the issue of managing hemodynamically unstable patients with limited and unspecific information.

In addition to this, it is believed that our fundamental understanding of cardiovascular physiology as encompassed by Guytonian models might be incomplete and insufficient under the clinical conditions encountered in peri-operative and critical care. Early experiments underpinning such models are still being debated [16,17]. At the core of Guytonian models is the interaction between cardiac function and the return of blood to the heart. This provides an important but simplistic construct for assessing hemodynamic (in)stability and response to therapy. The limited amount of information available via standard hemodynamic measures could be one of the factors limiting our ability to further apply our developing understanding of cardiovascular physiology in clinical practice and to expand existing models of potential value to peri-operative and critical care patients.

**Figure 1:**
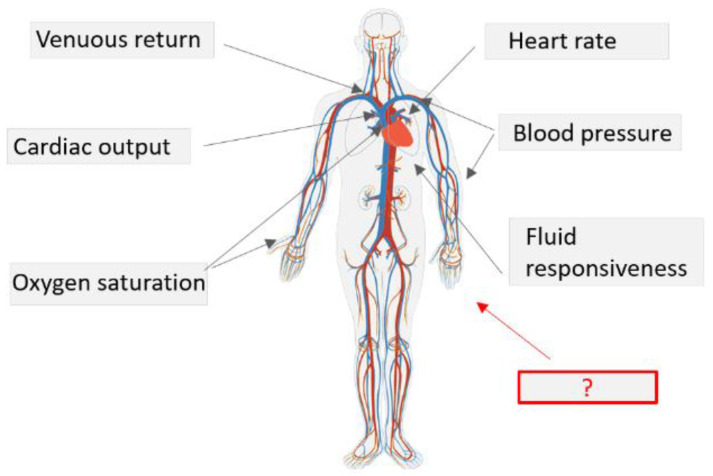
While work aimed at refining measurements of standard parameters is unarguably of great relevance for peri-operative and critical care, a pressing need exists for complementing such established parameters with additional hemodynamic indices. Such indices however are slow to find their way to standard clinical diagnosis and treatment procedures.

While the work aimed at refining measurements of standard parameters is unarguably of great relevance for peri-operative and critical care, a pressing need exists for complementing such established parameters with additional hemodynamic indices (Figure 1). This is important both for improvement of patient management in standard practice, as well as for further developing our fundamental understanding of the (patho)physiological processes that underlie cardiovascular complications.

A majority of efforts aimed at obtaining more complete information on the cardiovascular system via bed-side measurements rely on four main strategies:

1. Interpretation of the widely used parameters via functional hemodynamic monitoring. This is based on measuring the response of the circulatory system to a defined stimulus [1].
2. Measurement of the microcirculation [13]—direct assessment of the pathways of oxygen delivery and of the primary site at which oxygen exchange takes place.
3. Emerging technologies for characterization of dynamic vascular regulation—recent cardiovascular research is focused on the study of natural compensatory mechanisms. Changes in vascular properties (compliance, viscosity, and artery-vein interaction [18,19,20]) can precede changes in commonly measured hemodynamic indices such as BP, CO.
4. Data-driven, machine-learning (ML) assessment of physiological signals. Statistical methods are used to identify complex interactions between physiological signal characteristics to predict adverse hemodynamic events or to estimate variables that are not directly measured.

Focusing on these four main strategies, this review identifies key aspects, challenges, and the clinical value of measuring novel hemodynamic parameters in critical care.

A general pattern across the work carried out on this topic is that extensive developments are being realized in research, but the findings are slow to find their way to standard clinical diagnosis and treatment procedures. An analysis is needed to reveal different approaches and points of view from the perspectives of clinical research as well as technology/sensor development. By conveying the complexity of hemodynamic monitoring today as well as envisioned future directions, this review intends to provide insights into what is needed to accelerate the translation of research findings into meaningful implications for patient care.

## 2. Interpretation of the Widely Used Parameters via Functional Hemodynamic Monitoring

Functional hemodynamic monitoring consists of measuring the circulatory system’s response to a well-defined stimulus. While these approaches measure the same well-known hemodynamic parameters such as CO, BP, and SpO2, additional interpretation is given to the measurement by accounting for a controlled perturbation, offering extra information and a more complete picture of the status of a hemodynamically unstable patient [1,21]. For example, the passive leg raise technique, which has been the focus of many recent studies [22], measures CO with respect to a sudden blood shift towards the heart. The response of the CO parameter with respect to this blood volume shift reveals whether the patient is fluid responsive in the case of a suspected need for fluid administration.

Compared with static measurements, functional (dynamic) measurements can better reveal when compensatory mechanisms of the circulatory system are activated, thus enabling earlier detection of deterioration and proactive interventions.

Measurements already employed in some clinical settings are based on interpreting heart-lung interaction for the purpose of assessing fluid responsiveness. An example is the pulse pressure variation (PPV) parameter, which quantifies the changes in arterial pulse pressure during mechanical ventilation [23]. Other measurements include stroke volume variation (SVV), the end-expiratory occlusion test, and the tidal volume challenge. Some heart-lung interaction parameters can also be measured non-invasively via transesophageal echocardiographic approaches or via pulse contour analysis [24].

Other measurement strategies involve analyzing the response of the vasculature to cuff-based occlusions. Across applications, this principle has been used to investigate a multitude of conditions (e.g., venous thrombosis [20] and endothelial dysfunction [25,26]). In critical care, cuff-based perturbations have been used in combination with near-infrared spectroscopy devices [21,27] to obtain information on tissue metabolic function. In addition, the venous-arterial equilibrium pressure measured distally from the cuff post-occlusion is an indicator of mean systemic filling pressure [28].

Variations of the same concept can be adapted to a large number of monitoring settings. For example, the ECG signal morphology response to an increase in heart rate can be analyzed to reveal coronary artery properties [21], the arterial pressure response during a Valsalva maneuver can predict fluid responsiveness [29], and the microcirculatory function has also been studied with respect to thermal challenges [30]).

### Current Challenges

Functional hemodynamic monitoring has been gaining increasing interest from clinicians for its ability to enable more proactive patient management with existing clinical technologies. Especially when it comes to personalizing fluid management, dynamic measurements (PPV, SVV, passive leg raise, and fluid challenge) are recommended over combinations of static measurements (such as BP, HR, CO, urine output, central venous oxygen saturation, and lactate).

However, functional measurements share similar limitations with standard vital sign measurements. There are uncertainties regarding the interpretability of the measures; it is unclear how to achieve more personalized targets. For example, PPV is unarguably valuable for predicting fluid responsiveness. However, in some cases, a clear distinction between fluid responders and non-responders is difficult to define. For this reason, the spectrum of PPV values has been categorized into three zones: PPV values expected from responders, PPV values expected for non-responders, and a PPV value “gray zone” where a patient- and context-specific levels of uncertainty exist in the interpretability of the measure. (e.g., “Applicability of pulse pressure variation: How many shades of gray” [31]). This limitation is similar to the extensively studied difficulty of defining a threshold BP value to avoid hypotensive events.

Furthermore, similarly to standard measures such as BP and CO, a compromise exists between degree of invasiveness/obtrusiveness and ability to perform continuous/repeatable functional measurements. This compromise is particularly evident in stop-flow measurements, where a cuff is kept inflated for an extended period of time on a patient’s arm to reliably measure dynamic tissue oxygen saturation. Furthermore, the usefulness of PPV and SVV is questioned when lung-protective, low-tidal volume mechanical ventilation is used [32].

In addition to this, functional measurements pose extra challenges compared with standard measurements, mainly due to their novel nature. For example, the passive leg raise involves bed adjustments and possibly other actions that might differ from the standard clinical workflow of managing the general patient population. The passive leg raise is generally limited to particular patients and clinical cases, e.g., it is typically performed to assess fluid responsiveness prior to resuscitation for the already hemodynamically unstable patient. Other well-known functional parameters such as SVV/PPV are mainly intended for the invasively monitored, mechanically ventilated patients in deep sedation, thus limiting applicability to the OR/ICU [23]. Functional measurements are not yet standard for prediction/detection of adverse events in the general patient population (for example, in the wards). Automation or the inclusion of functional measurements in the standard detection of deterioration spot-check might be found impractical in the near future.

## 3. Develop Technology Specifically Designed to Assess Microcirculation

Inadequate tissue perfusion results in insufficient oxygen supply to cells. One way of identifying alterations in the supply of oxygen is to assess the microcirculation, the primary site at which oxygen exchange takes place. Significant regulation mechanisms occur at the microcirculatory level, and this was first observed via direct visualization of the capillaries [33]. Parameters such as capillary density, heterogeneity of capillary distribution, and proportion of stopped or intermittently perfused capillaries were found to be linked to various conditions. For this reason, in the past three decades, hand-held microscopy at the bedside has undergone major technological advancements to allow for an in-depth study of the microcirculation. By overcoming device limitations (such as motion artifacts, calibration requirements, measurement depth, temporal resolution, automated feature extraction, and device bulkiness), it was possible to assess the relevance of measurement locations and the specificity of different morphological parameters. Visualization of the pathways of oxygen delivery improved the understanding of tissue oxygenation, capillary alterations in relation to the progression of disease, the pathophysiology of septic, cardiogenic, and hemorrhagic shock [34], and expected patient outcomes [35,36].

In parallel to the development of direct capillary visualization, other optical technologies such as near-infrared spectroscopy were adapted to measure tissue oxygenation as a surrogate for microcirculatory function [37,38]. These technologies have been found particularly relevant in the assessment of cardiovascular reserve and tissue metabolic function via analysis of reoxygenation/deoxygenation rates in relation to occlusion-based perturbations. Interesting physiological [39] and pathophysiological [21] mechanisms have been observed via measurement of tissue oxygenation responses to vascular occlusion tests. 

### Current Challenges

Despite their significant contribution to advanced understanding of regulation mechanisms, the devices designed for measurement of microcirculation face a range of challenges in terms of inclusion in clinical hemodynamic monitoring practice in the general patient population [40]. The measurements are very localized and only reflect the quality of the microcirculation at pre-defined sites. For example, it is not known if sublingual microcirculation reflects other sites, such as the gut [41]. Furthermore, peripheral locations such as the limbs might be influenced by factors such as temperature, which can impair the interpretability of microcirculatory measurements. It is not yet clear how to make use of existing microcirculation measurements for prediction of hemodynamic instability or therapy guidance. Ongoing research aims to solve uncertainties around microcirculatory response to systemic macrocirculatory changes [12] and differences between measurement sites in the body [42,43,44]. There are remaining questions that still need to be answered to achieve standardization, and formal technology assessments are required to determine the precise role of these devices in critical care [33,45].

Inevitably, further development of optical measurement techniques will take place to enable observations related to the structural and functional characteristics of the microcirculation. Optical methods that are currently being investigated involve technologies such as laser Doppler imaging, laser speckle contrast imaging [46], and optical coherence tomography [47]. It is argued that optical measurements are limited to easily accessible surfaces, such as skin, muscle, and tongue [42], and therefore, it is unlikely that optical measurements alone will achieve a comprehensive understanding of microcirculatory alterations (such as in different organs) [33,42,48].

“Despite their high spatial and temporal resolutions, optical imaging modalities are still not widely used for clinical imaging of the microcirculation due to their limited tissue penetration. Diffuse optical imaging techniques such as NIRS ameliorate this to some extent, but at the expense of spatial resolution.”

[48]

For this reason, microcirculatory measurement devices intended for hemodynamic monitoring will likely involve optical methods complemented by other technologies such as ultrasound (US) and magnetic resonance imaging (MRI). Recent studies have made use of US to identify renal microcirculation alterations [49], and hybrid technologies involving photo-acoustics are currently being investigated [50]. Such studies, together with novel developments of thin-film, wearable ultrasound transducers [51], may open up new possibilities for bed-side measurement of microcirculatory function. In addition to this, insights acquired from applications such as coronary disease management [52] will likely serve as the basis for applying MRI to better understand oxygen delivery mechanisms in different organs and under different pathological states.

## 4. More Advanced Assessment of the Vascular Properties

### 4.1. Cardiovascular Research and Its Relevance for Critical Care

Cardiovascular diseases are a leading cause of death worldwide. Global efforts are aimed at understanding cardiovascular disease prevention, treatment, and management [53]. Emerging insights have contributed to the development of models, devices, measurement procedures for cardiovascular indices, therapy standardization, as well as the discovery of new research trajectories. The solutions developed for cardiovascular disease management have enabled understanding of the arterial system, including the arterial wall-blood interface, coupling between the heart and vasculature, and local and systemic arterial properties.

In particular, significant evidence on the importance of active arterial regulation mechanisms has been found. Studies are revealing the complex, dynamic behavior of arteries on local as well as systemic levels [19,54,55]. Since early observations on the ability of vessels to constrict/distend, evidence about other mechanisms has been found: pulse propagation, systemic resistance, local flow, and wave reflection are controlled via factors such as the non-linear properties of the arterial wall, viscosity, damping properties, frequency-dependent behavior, and strain-dependent activation of the smooth muscle tone [19]. Due to this, a more integrative approach to physiology is being encouraged nowadays, in which vascular compensatory mechanisms are studied. Methods for observing and measuring these compensatory mechanisms are being developed.

“When modeling blood circulation, the heart is usually considered the main element, the only one which has an actual relevance in the operation of the system, thus neglecting blood vessels, which are considered simple conduits that connect the cardiac pump with the organs. Such a basic approach underestimates the prominent role shown by blood vessels in general and by arteries in particular“, “The active stress developed by smooth muscle has been overlooked as a contributor to the mechanic behavior of vessels, although it has been demonstrated that the activation of smooth muscle changes the stress–strain relationship towards high levels of stress”, “This is very important when studying the cardiovascular system given the active participation of the nervous system in hemodynamic regulation.”

[19]

Short-term, the dynamic regulation of arteries is especially relevant in hemodynamic monitoring. Therefore, it is worth investigating how to adapt the recent findings in cardiovascular disease management to overcome limitations in patient monitoring. Changes in vascular properties can precede changes in commonly measured hemodynamic indices such as BP and CO.

Given that advances in the field of cardiovascular disease management are not usually discussed in the context of critical care, this section will require a more complex overview. For this reason, this section has been subdivided to allow for the inclusion of more in-depth background information related to cardiovascular disease management parameters and their potential relevance for hemodynamic monitoring.

### 4.2. Arterial Stiffness

Up until now, extensive studies on arterial stiffness have been conducted. Many of these studies are aimed at understanding the strong link between the level of distension of the arteries and BP, with the aim of developing continuous, non-obtrusive (cuff-less) BP measurements. Beat-by-beat BP fluctuations throughout the day as well as long-term trends in BP over years can reveal the impact of lifestyle and pathological conditions on cardiovascular function. The most promising solution for monitoring BP continuously and non-invasively involves measurement of arterial stiffness (or compliance).

“An important overarching concept underlying cuffless measurement of blood pressure is the fundamental relationship between transmural pressure and mechanical properties of the arterial wall which influence wave propagation phenomena. This is the pressure dependency of the material stiffness of all blood vessels. This property is present in all species with pressurized circulatory systems and is a fundamental evolutionary property of arterial design.”

[18]

The level of distension of the arteries can be deduced from studying the speed at which the wave propagates along an artery (pulse wave velocity, PWV, pulse transit time, and PTT), or by studying the artery volume amplitude/waveforms. Since arterial stiffness is influenced by blood pressure, it can thus be assumed that a change in wave propagation is caused by a change in BP. For this reason, a large number of technologies have been designed to acquire information on PWV/arterial distension (ultrasound, tonometry, radar, photoplethysmography, phonocardiography, impedance cardiography, seismocardiography, electrical impedance tomography, and ballistocardiography [18]).

“PTT is a measure for arterial stiffness. When blood pressure increases, the vascular tone increases and the arterial wall becomes stiffer, causing the PTT to shorten.”

[18]

However, major limitations have been identified, such as the uncertainty of arterial stiffness being influenced by other factors besides BP (e.g., smooth muscle activation via hemodynamic regulation mechanisms) and the difficulty in approximating the BP value—surrogate value relationship (e.g., a change in wave propagation speed of x ms reveals a change in BP of y mmHg). Frequent re-calibrations by means of simultaneous measurement of BP and PWV can in principle solve such issues. Initialization algorithms are also employed to reduce the number of obtrusive re-calibrations or increase the accuracy of the estimates. Solutions based on empirical knowledge and physiological models combined with demographic data (age, sex, weight, and height) have been attempted. Despite the various proposed solutions, at the moment there is still no clear optimal method for linking PWV to BP, as many other items remain unsolved: location for surrogate measurement, distance travelled by pulse, inability to assume artery uniformity over large distances, peripheral arterial beds being significantly affected by regulation mechanisms, re-calibration intervals etc.

“Is “Cuffless” the Future of Blood Pressure Monitoring? There have been many patent submissions, start-up companies, and scientific publications, but to date there is no device that is universally accepted by the wider community beyond research laboratories and company boardrooms.”

[18]

#### 4.2.1. Insights on Vascular Regulation Mechanisms

Even though the non-obtrusive or minimally obtrusive BP measurement is still considered an open question, the large-scale nature of the research resulted in the collection of extensive data related to arterial properties, regulation mechanisms, and vascular hemodynamics. For example, PWV and arterial volume waveforms have been studied in the context of exercise, rest [56], sleep [57], or the administration of vasoactive medication [58,59]. Studies have also been conducted to understand arteries at local sites (brachial, radial, and finger site) vs. regional sites (PWV measured from heart to finger, or heart to femoral, toe, or combinations) and peripheral vs. central regulation [60]. Other studies are dedicated to the analysis of PWV as a function of different mean lumen diameters [61]. Measurements involving pulse arrival time (PAT) or pulse transit time (PTT) confirm long-standing data that smooth muscle is sparser at the aorta, while in smaller arteries, smooth muscle can contract enough to impact pulse transit independently from BP [62]. PAT/PTT models have also revealed interesting processes of the circulatory system, such as blood redistribution during digestion, in the context of ambulatory BP measurements [63,64]. This is relevant for identifying when to trigger a recalibration of the BP value—PAT surrogate relationship.

#### 4.2.2. Insights on Technology Requirements

Besides aiding in making observations on regulation mechanisms, the large number of studies has also contributed to identifying technology requirements for measurement of arterial properties. Device characteristics such as PPG sensor contact impact on PTT [65], hydrostatic effects [66], BP cuff size/shape/design properties [67,68,69], different cuff inflation strategies [60,70], and measurement of cardiac factors [71] have been investigated, and processes for validating devices have been improved [66]. Motion artifacts and signal-to-noise ratio have also been thoroughly investigated [72]. Several cuff and cuffless device prototypes have been built, and many measurement strategies have been tried out to sample arterial beds. Some examples involving variations of the cuff, ECG, PPG, and tonometric technologies are summarized in Table 1.

In addition to prototypes aiming to improve, adapt, or re-interpret typical cuff, ECG, PPG, and tonometry technologies, several research groups and start-ups have attempted out-of-the-box measurements, also providing unique technology insights—glasses with PAT sensors [80], BP measurement underwear [81], weighing scale BP estimation involving ballistocardiogram and PPG signal measured from the sole of the foot [82], other wearables [83], phone solutions [84], elaborate measurement settings involving technologies such as electrical impedance tomography [85], contactless transdermal optical imaging [86].

#### 4.2.3. Value for Critical Care

It is important to assess whether the arterial tone can be re-interpreted for assessing the short-term adjustments in arterial properties for applications in critical care. Knowledge of smooth muscle tone (arterial compliance/stiffness) could be of high clinical interest, as will be outlined below. ICU clinicians have expressed interest in these parameters [6], with vascular compliance having a significant impact on CO estimations. However, measurements of arterial compliance in critical care are scarce. Methods for quantifying and interpreting short-term, dynamic regulation of arteries have yet to be investigated and developed.

##### Value for Recalibration of Standard Measurements

As a first application in critical care, arterial stiffness estimates can be used for measurements requiring calibration. Devices that use pulse waveform as a surrogate of cardiac output (PiCCO) require re-calibration whenever changes in arterial compliance occur as a result of hemodynamic regulation mechanisms, vasoactive drug administration, or in cases of the development of vascular edema [6].

“The major weakness of all these devices is the drift in values whenever there is a major change in vascular compliance, as, for example, in vascular leak syndrome with increased vessel wall edema leading to decreased arterial compliance.”

[6]

Therefore, identifying when such devices require re-calibration can be a first relevant application to introduce arterial property measurements in clinical hemodynamic monitoring practice. Such applications can serve as the basis for developing a better understanding of arterial regulation and tackling other unmet clinical needs.

##### Value as Predictive Parameter and Improvement of Blood Pressure Estimation

Changes in arterial properties might precede changes in blood pressure and flow and may be used for early warning of hypotension and hemodynamic instability. Moreover, information on arterial compliance can aid in the interpretation of blood pressure values. Complementing the BP value with more information could make the measurement more specific.

For example, arterial compliance measurements can be used in principle to differentiate between heart-related factors or vasodilation to identify the cause of a BP drop, thus facilitating improved fluid management. In addition to this, insights on the dissociation between BP (or other macrocirculatory variables) and microcirculation could possibly be acquired from assessments of arterial compliance at central vs. peripheral vascular beds.

Another application likely to benefit from the recent findings related to arterial compliance is the accuracy of cuff-based non-invasive BP measurement (NIBP). NIBP is still based on an empirical interpretation of the cuff pressure signal, which leads to large systematic errors in BP estimations, especially in cases of hypotension and hypertension. An in-depth characterization of the vasculature response to occlusion-based perturbation (such as cuff-induced arterial collapse and resulting effects on pulse propagation) can lead to a better understanding of the cuff measurement principle, possibly leading to more accurate NIBP [65,87].

#### 4.2.4. Current Challenges

Despite the plethora of health data related to arterial properties available for interpretation, development is still needed for the implementation of arterial compliance measurements in critical care. Concrete findings and standardized measurement setups are still lacking, and controversy exists in the community. In many cases, the studies involving PAT/PTT are performed on small numbers of subjects, allowing for interesting qualitative insights, hypothesis identification, and the discovery of new research directions; at the same time, the scarce nature of the measurements and variability in non-standardized measurement setups lead to uncertainty. There are evident and exhaustively discussed conflicting results regarding the usage of PAT for BP monitoring [88,89,90]. As another example, the study of vasoactive drug effects on specific vascular beds is also not conclusive [58,59]. Zong et al. [58] reveal that vasoactive drugs do not influence arterial wall elasticity in the arm, while Banks et al. [59] conclude the opposite—significant smooth muscle relaxation at the brachial site is measured after administration of vasoactive drugs. However, the two studies are realized via very different methods—in the work by Zong et al. [58], the PAT is measured over a large distance via ECG and the ABP (heart to radial site) is analysed in ICU patients. In the work by Banks et al. [59], localized ultrasound imaging is employed to measure artery size at the brachial site in healthy subjects.

Another example of unresolved conflicting findings is the effect of PPG contact pressure on finger vasculature. In two studies [65,91], different findings are reported about the shape of PAT as a function of PPG contact pressure, and it is recognized that the difference in the findings cannot yet be explained.

Comparing seemingly disagreeing studies or interpreting individual studies within a bigger picture is difficult to accomplish without the existence of standardized databases to validate assumptions, account for error sources, and validate signal processing algorithms (e.g., the MIMIC database). Many aspects of arterial compliance are currently being investigated [56,57,58,59,88,89,90,92]; further studies are required to converge the various research modalities and findings.

“The direct effect of smooth muscle relaxation on arterial elastic properties is controversial.”” In human subjects, the contribution of smooth muscle to arterial elastic mechanics has been limited by difficulty in separating the direct effects of a vasodilator drug on the arterial wall from the indirect effects due to reduced blood pressure.”

[59]

More dedicated studies are required for the reinterpretation of cardiovascular disease research in the context of hemodynamic monitoring. Preliminary, on-going research is tackling the challenge of establishing a standardized setup and overcoming some of the uncertainties of patient monitoring devices [93]. The setup presented in [93] aims to reduce the variability in existing BP cuffs and to determine whether compressibility of the arm due to cuff inflation is a significant source of error in cuff-based arterial compliance measurements. Besides this, a measurement setup involving existing hemodynamic monitoring devices (ECG, PPG, and BP cuff), together with a dedicated signal processing algorithm, has recently been proposed for measurement of arterial stiffness in critical care [75], and feasibility has been demonstrated via computer simulations and limited patient data.

### 4.3. Beyond Arterial Stiffness

An example of a cardiovascular research topic to be reinterpreted for critical care applications is the detection of venous thrombosis [20]. In ref. [20], a parametric hemodynamic model describes the interaction between arterial and venous beds in partially occluded limbs; the model parameters are representative of clinically relevant indices related to thrombosis. The model, however, also includes parameters such as systemic resistance and blood vessel compliance, which are also relevant to hemodynamic monitoring, as stated by the author:

“Finally, it is particularly important to note that changes in the compliance and resistance deduced with the aid of the model exhibit a dependency on pressure and flow, respectively, which is characteristic of the compliance and resistance of blood vessels. This suggests that a particularly appropriate application for the model is to use changes in the model parameters to monitor circulatory changes of the limb, such as those, for instance, that may occur during clinical anaesthesia.”

[20]

**Figure 2:**
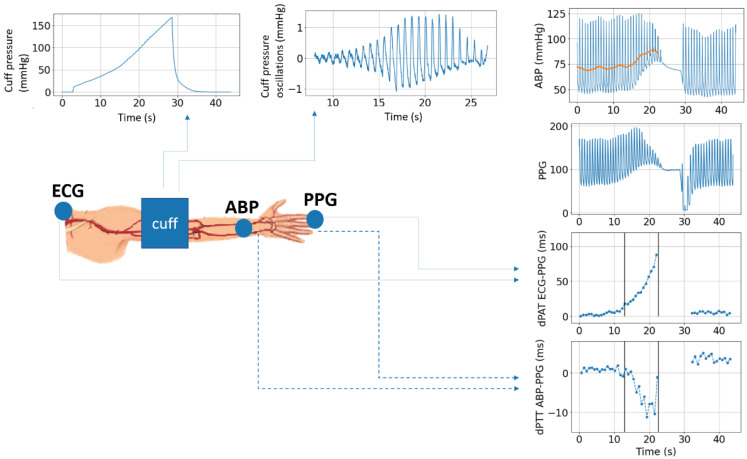
The vasculature response to cuff inflation is complex, highly dynamic mechanisms are observed via invasively acquired BP signal (ABP), ECG and PPG based measurement of PAT/PTT across different segments of the limb. Such mechanisms are informative of patient status can be characterized via cardiovascular models to infer a number of parameters relevant to hemodynamic monitoring [94].

To further investigate this, in ref. [94], an adaptation of this model has been used to study data acquired in the OR/ICU. The response of the vasculature to cuff-based perturbation is measured via standard monitoring equipment (BP cuff, ECG, PPG, intra-arterial line), and a model similar to [20] is used to interpret the behaviour observed in the vasculature located under the cuff and distal from the cuff during a typical cuff inflation as performed via standard NIBP. It was shown that complex dynamics occur in the vasculature during cuff inflation (as illustrated in Figure 2). Such responses can be processed to estimate informative parameters related to pulse arrival time vs. BP calibration, artery compliance, peripheral resistance, and artery-vein interaction. The main conclusion the study conveys is that standard hospital equipment is largely underutilized and that various arrangements of the standard sensors can achieve a more complete assessment of the patient’s status, for example via the interpretation of the vasculature’s response to cuff-based perturbations.

The aim of the studies conducted in refs. [20,94] is to characterize the vasculature’s response to partial occlusions, or to occlusions of short duration. The intention is to perturb the circulation as little as possible in order to avoid activation of regulation mechanisms (e.g., arterial compliance and peripheral resistance are not altered as a result of the cuff inflation). There are, however, other categories of cuff-based measurements that do in fact aim to alter such parameters by means of occluding the artery for approximately 5 min, or more. In the case of endothelial function assessment [25,95], ischemia is induced in the limb; compliance and resistance of the distal vasculature are altered as a result of the reduced pressure in the distal artery during occlusion and the subsequent increase in flow post-occlusion. Changes in arterial dimensions reveal the extent to which regulatory mechanisms are activated in response to tissue hypoxia. A standard method, flow-mediated dilation (FMD), is used to assess vascular dysfunction by measuring the artery diameter increase in response to reactive hyperemia induced by occluding the artery for approximately 5 min.

Such measurements are well-established in the assessment of cardiovascular health, and extensive efforts are aimed at further developments to enable standardization across disciplines, including critical care [26,27].

Another example of relevant research involves the study of the non-linear properties of arterial collapse [19,78,96,97,98]. Such properties are studied by estimating arterial cross-sectional area or compliance under different transmural pressures across the arterial wall. There is interest in finding whether such non-linear vascular parameters adapt due to vasoactive medication or due to activation of regulation mechanisms.

Many other regulation mechanisms linked to viscosity, damping properties, and strain-dependent activation of smooth muscle tone are being investigated [19,99,100]. Such dynamic behaviors can be studied at local as well as systemic levels [101,102].

The studies give an outlook on the large amount of research still needed to characterize the intricate mechanisms of the cardiovascular system. Such research is currently difficult to perform in-vivo; further developments are enabled by rapid technological advancements, such as the continuous measurement of vascular flow and diameter by means of wearable, operator-independent ultrasound patches [103,104].

#### Current Challenges

Modelling “beyond arterial stiffness” is important from a research perspective for obtaining physiological understanding. However, limitations exist in terms of clinical implementation; it is recognized that very in-depth modelling is unlikely to have practical applicability since the circulatory system is far too complex and patient/context specific. Accurate measurement of many parameters, such as pressure, flow waveforms, and pulse travel across arterial segments, might not be realistic in standard patient care.

“It is well known that hemodynamics of large arteries is too complex to be apprehended using only non-invasive measurements and medical imaging techniques.”, “As 3D models can only be used in small portions of the cardiovascular system due to their high modelling and computational costs, reduced-order models have gained attention to reproduce complex wave propagation behaviors in large networks of arteries.”, “Although arterial pressure is easy to measure, the precise measurement of blood pressure requires highly invasive techniques.” [19] “In cases where it is not possible to develop physical models it becomes necessary to use shortcuts based on empirical, statistical, or even simple profile models.”

[105]

## 5. Data-Driven Approaches

The arterial pulse signal contains ample information regarding complex homeostasis processes, and this knowledge has been documented as early as the 2nd century [106]. In traditional Chinese and Greek medicine, the pulse is seen as an important source for understanding health and disease. The reading of the pulse has always been recognized as having potential for aiding medical practice. First observations were based on a qualitative assessment on rhythmicity, pulse variation, and the elasticity of the artery.

“How can one tell whether a pulse is ‘full’, ‘rapid’ or ‘rhythmical’? Is there a perceptible pause when the artery has reached the limit of its contraction and, again, of this expansion? Is there also a pause when the artery returns to its normal size? Such questions Galen resolved partly historically, by referring to earlier authorities, and partly from experience. His own enthusiasm for studying the pulse, which has been with him since youth, and his hours of practice had given him, he claimed, a most sensitive touch, an example worth imitation.”

[106]

Today, advances in sensor technology and physiological models have allowed for the development of reliable HR, BP, and blood oxygenation estimations based on pulse measurements. In some cases, measurement of CO and SV and estimation of some arterial properties are also possible via the pulse, although such measurements come with several assumptions and controversy.

It is evident that the pulse contains far more information than is currently exploited; lately, data-driven approaches have been used to attempt extracting this additional information from the pulse. Such methods are based on statistics and have the potential to pick up on subtle signal characteristics in large datasets while bypassing the need for circulatory system physiological characterization.

As an example, the Hypotension Prediction Index algorithm (HPI; Edwards Lifesciences, Irvine, CA, USA) uses supervised learning to process high-fidelity arterial pressure waveforms and to estimate the likelihood that a hypotension event will occur 5–15 min in the future [107]. The algorithm is trained to detect which interactions among thousands of potentially informative waveform features reveal compensatory mechanisms preceding a drop in blood pressure. The aim is to use the HPI and related information to act before hemodynamic instability occurs.

A great variety of other ML methods have been applied to process information encoded in signals acquired via PPG, ECG, intra-arterial lines, cuffs, and volume clamp sensors. In fact, most of the existing ML algorithms have been adapted to process such physiological signals: variations of regression approaches, decision trees, support vector machines, and different types of neural networks [108,109,110]. Methods such as regression models and decision trees require complex handcrafting of potentially informative features, while methods based on neural networks are usually applied when minimal signal pre-processing takes place. In the latter case, the algorithm automatically discovers features from the data. 

Such ML techniques are designed, for example, to predict deterioration events, such as sepsis, hemorrhage, and unplanned intubation [111], or to estimate variables that are not directly measured (e.g., BP estimation via PPG/ECG waveform analysis [109,112]). It is not clear at this point which of the ML algorithms are most suitable for such applications. For this reason, some studies include thorough comparisons between the performance of different statistical methods [113]. 

However, the choice of algorithm might play a secondary role in comparison to other factors, such as defining the information source that is most relevant for a given application. For example, in ref. [107], the relevant information source for prediction of hypotension is defined as the pulse waveform—subtle adjustments and interactions in waveform features are identified when changes in compensatory mechanisms take place. In ref. [113], however, the algorithm for prediction of hypotensive events does not take waveforms into account, arguing that the resulting features are too reliant on signal quality (waveform quality depends on hardware, filtering, and digital signal processing). Instead, minimal information regarding the pulse (e.g., absolute values of vital signs) and ample context information (medications, ventilator data, demographics) are defined as relevant information sources. Both approaches are considered promising [114].

### 5.1. Current Challenges

Despite the large number of identified opportunities, extensive research, and great variety of investigated algorithms, ML has not yet been established as a standard clinical tool in hemodynamic monitoring [115]. In other fields, ML algorithms are found to be as beneficial as theoretical models, and in some specific applications, ML algorithms are found to even surpass the performance of conventional methods for signal processing and analysis. For example, deep learning has clearly surpassed standard approaches in image processing segmentation tasks [116]. Furthermore, ML-based reconstruction of MRI images can be achieved by entirely bypassing the inverse Fourier transform, opening the door to interesting developments in image acquisition strategies [117]. There are several factors that could explain the limited usage of ML in hemodynamic monitoring.

One such factor is the difficulty of accessing good quality data due to the variety of measurement setups and medical procedures, the dependency on operator skills to place sensors, the transport of patients which usually involves changes from one recoding system to another (e.g., OR to ICU to ward). Furthermore, in many cases, it is difficult to document procedures and patient history or outcome. It is still unclear how to fully document the context.

In addition to this, many sensor effects are not fully understood or characterized. PPG contact pressure, cuff size and placement, and wrapping tightness have recently been found to affect signal quality and interpretability [65,67,68,69,72]. In certain cases, even sensor effects of invasive measures such as arterial lines can make the data unusable (e.g., inaccurate damping). Waveform signal quality depends on hardware, filtering, and digital signal processing, if these steps are not known in depth, then ML methods can be unreliable. Besides sensor effects, physiological effects are also not fully understood. Tissue compression and pressure transmission effects in cuff inflation [93] or volume clamp devices are particularly difficult to account for in ML models, which lack transparency and interpretability [118]. Improved standardization of the measurement procedures is needed to enable data-driven methods.

In terms of databases available for algorithm training, in machine vision applications, the performance of deep learning is strongly dependent on large amounts of annotated images collected over years. This might not be as feasible for 1D signals. Manual annotation is more difficult for 1D signals, and few clinicians are available to complete annotation tasks. For example, in radiology, clinicians annotate X-ray images by default; in hemodynamic monitoring, it does not occur in regular practice to annotate PPG/ECG/cuff signals in detail.

Even in the case of a well-annotated database, human biology and pathology can vary considerably. Applying ML to find the inverse function of a sensor vs. applying ML to understand physiological data are very different tasks. It is difficult to assess whether a database is sufficiently rich to represent the full variability of the underlying pathophysiology. Most existing algorithms are developed based on single-center studies. For this reason, algorithms usually work well on retrospective data, or in controlled environments, but they fail when they are applied to new patient groups or contexts. This poses a fundamental problem of the generalizability of the results and obtained ML models. For example:

- Cuff-based non-invasive BP measurement algorithms are optimized for normotensive patients, and significant errors in clinical BP readings are being reported in hypo- and hypertensive patients [119].
- An algorithm for predicting kidney injury (DeepMinds [120]) was trained on data collected mainly from patients in a veteran hospital—a demographic that is not representative of the general population.

Another aspect is that, in most cases, defining a well-posed problem suitable for ML interpretation in hemodynamic monitoring is difficult. For example, is hypotension defined at 60 mmHg, 65 mmHg, or personalized for every patient? [9–12,121].

Ultimately, there is no clinical interest in simply predicting parameters such as BP. ML is only relevant when data can be linked to actions that are relevant for clinical practice, adding a level of complexity compared with other fields where ML has been used.

ML methods are not yet standard in clinical practice [110]. While it is known that the information contained in arterial pulsations is underutilized, it is still unclear to what extent additional information can be extracted via ML.

### 5.2. Future Possibilities Involving Data Infrastructures and Explainable Artificial Intelligence

There have been efforts aimed at increasing the quality of ML algorithms via data infrastructures across research centers. The focus is placed on standardized collection and annotation of data, facilitating re-utilization of databases, and the validation of algorithms. Other parallel work is aimed at overcoming hemodynamic monitoring sensor limitations to allow for more reliable recording of hemodynamic signals. In some cases, ML itself is used to solve for sensor limitations, for example, in overcoming inter-operator variability and motion sensitivity in ultrasound image acquisition [104].

Innovation in the field of big data will also likely enable the development of frameworks compatible with different information sources, such as complex pulse waveform data as well as patient- and context-specific information. This is relevant, especially in view of the large variety of emerging technologies aimed at measurement of microcirculatory properties, vascular response to controlled perturbations, and regulation mechanisms linked to elasticity, viscosity, and smooth muscle characteristics. Innovative frameworks aimed at the aggregation of large amounts from information will be necessary to identify correlations between parameters and develop improved hemodynamic models.

At the same time, new perspectives on the development of more ‘interpretable’ ML models [122,123] might play a role in the future applications of artificial intelligence for hemodynamic monitoring. Furthermore, the increasing interest in the concept of ‘digital twin’ across fields of research might help re-evaluate the way ML algorithms are applied to understand physiology.

## 6. Discussion

This review presented current trends and main outlooks for advances in critical care via measurement of new hemodynamic parameters: a reinterpretation of the widely used parameters via functional hemodynamic monitoring (measuring response of the circulatory system to defined stimuli), technology aimed at observing the microcirculation, adapting knowledge acquired from cardiovascular disease research, and machine learning approaches. All four identified research directions are well-founded, and they unarguably stimulate new, innovative research that is essential to advancing our understanding of vascular physiology.

However, the four research directions do differ in terms of their near-future impact on standard hemodynamic monitoring practice. Several aspects need to be taken into account in order to achieve effective translation of technology into clinical practice. On a practical level, it is important to consider the extent to which the proposed equipment differs from existing hospital equipment, the dependence on the operator, standard practices, and staff training [124]. “*The major issue going forward will not be the sensitivity or specificity of any new index, since most are quite sensitive and specific, it will be the ease to which they can be assessed continuously or repeatedly and their level of invasiveness.*” [21]. On a more fundamental level, great difficulty comes from the challenge of evaluating the performance of a hemodynamic monitoring system. Access to additional hemodynamic parameters might not guarantee an improved patient outcome, and the process of assessing the usefulness of advanced monitoring is complex [125]. “*no monitoring tool, no matter how accurate, by itself has improved patient outcome*.” [1], “*Hemodynamic monitoring systems are measurement tools and their effects on outcomes are only as good as the protocols they are used to drive*.” [125]. It is likely that relatively slow, incremental changes to existing medical equipment and protocols will take place to allow for such an evaluation of their usefulness. A measurement strategy that does not differ to a large extent from the standard practice is arguably easier to include in large-scale extensive clinical studies designed to assess the impact of the monitoring system.

Therefore, ideal solutions for near-future implementations will be based on standard, existing hospital equipment [124]. Due to this, functional parameters and several parameters adapted from cardiovascular research will be more immediately investigated in a clinical setting.

Regarding longer-term advances, the large-scale, world-wide effort aimed at understanding cardiovascular diseases combined with improved solutions for microcirculation assessment will point towards a much more complex analysis of the vascular system (e.g., viscoelastic effects, inclusion of capillary activity, artery-vein interaction). In terms of research, machine learning, big-data statistical tools will likely be employed to help synthesize the large amounts of measurements and context information [126], helping to achieve a more in-depth understanding of the circulatory system and the dynamic interactions between different hemodynamic parameters.

Exceedingly complex modelling of the arterial system is unlikely to have practical applicability beyond research. The circulatory system is far too complex, patient and context-specific, requiring highly invasive measurements for accurate, real-time characterization. For this reason, big-data tools will also help in determining a finite but optimized number of measurements and intelligent combinations of sensors that are most informative, safe, and practical for patient monitoring in clinical practice. Relatively simple but well-understood theoretical structures, aided by empirical evidence, will be used to interpret such measurements in clinical decision-support.

## 7. Conclusions

Hemodynamic monitoring has developed greatly over the last decade; many new technologies are becoming available in the clinic, and the measurements are safer and more accurate. However, the new devices are designed for improved measurement of the commonly used indices (e.g., BP, CO). Extensive research is aimed at obtaining a more complete picture of the hemodynamic status of a patient: (i) vasculature response to controlled perturbations has been demonstrated to reveal fluid responsiveness, mean systemic filling pressure, artery/vein compliance, tissue metabolic function, and various complex dynamic interactions among hemodynamic parameters; (ii) new sensors are being developed to allow for direct measurement of previously inaccessible microcirculatory parameters; (iii) extensive progress achieved in the field of cardiovascular disease management provides strategies for assessing parameters related to compliance, viscosity, and resistance of various vascular beds. (iv) machine learning inherently extracts ample information from hemodynamic signals; this information can be integrated within parameters/indexes which predict hypotension or reveal the likelihood of kidney injury.

However, such developments are slow to find their way to clinical practice; this review analyzed the unique and complex challenges that arise when attempting to include new hemodynamic parameters in standard practice.

Any progress requires strong interdisciplinary collaboration and the involvement of multiple medical specialties.

There is a need to complement technological/sensor advancements with improved standardization of measurement setups, the development of big data frameworks compatible with different information sources, and larger clinical studies. However, more fundamental questions might need to be tackled in parallel, such as how to evaluate the usefulness of a hemodynamic monitoring system and how to interpret new hemodynamic information with reference to existing models encompassing the current understanding of cardiovascular physiology.

## Figures and Tables

**Table 1:** Examples of technologies involving variations of cuff, ECG, PPG, tonometric technologies.

Device	Measurement Procedure
Arteriograph (TensioMed, Budapest, Hungary) [73]	A cuff placed at brachial site is pressurized suprasytolically, therefore removing effects stemming from vasculature under the cuff and distal from the cuff. Arterial pulsations occur at the upper edge of the cuff. The resulting cuff signal is processed to obtain information such as central pulse wave velocity, aortic pressure values, wave reflection characteristics.
Mobil-O-Graph (IEM Gmbm, Stolberg, Germany) [70]	A cuff placed at brachial site is used; the cuff pressure is recorded with the use of a high-fidelity pressure sensor. The inflation process includes several seconds where the cuff pressure is held at diastolic vale. The waveform is analyzed to estimate aortic pressure values, wave reflection characteristics.
Complior (ALAM, Vincennes, France) [74]	The method uses two non-invasive tonometric sensors to simultaneously record pulse waves in the carotid and femoral arteries to measure carotid-femoral pulse wave velocity for assessment of arterial stiffness.
PTT-BP calibration based on cuff modulation [75,76,77]	A cuff is used to alter transmural pressure across the brachial artery for the purpose of modulating PTT. The change in PTT with respect to the controlled transmural pressure is measured via ECG and PPG and analyzed in order to calibrate the BP-PAT relationship for the purpose of beat-to-beat PAT-based BP estimation and arterial stiffness estimation.
Chronos TM-2771 (A&D Company, Tokyo, Japan) [69]	Arterial diameter at brachial site is measured via a device consisting of four adjacent cuffs. The cuffs are designed of soft and hard materials such that the resulting cuff pressure oscillation reflects brachial artery volume oscillation as accurately as possible.
Water-filled cuff [78]	A water-filled blood pressure cuff is used to allow the brachial artery to be simultaneously imaged via ultrasound. The aim is to obtain an accurate measurement of brachial arterial volume during cuff-based occlusion.
SphygmoCor (AtCor Medical, Sydney, NSW, Australia) [79]	Brachial and femoral cuffs or, alternatively, applanation tonometry of the carotid and femoral sites are used together with specially developed algorithms to acquire central aortic pressure waveform and carotid-femoral arterial PWV for the purpose of estimating cardiovascular risk.
Aktiia SA (Aktiia SA, Neuchâtel, Switzerland) [66]	Optical sensors perform green reflective photoplethysmography (PPG) measurements on the skin vasculature of the wrist to measure BP.

## Data Availability

Data is not publicly available due to privacy restrictions.

## References

[1] Pinsky M.R., Payen D. (2005). Functional hemodynamic monitoring. Crit. Care.

[2] Aya H.D., Cecconi M., Pinsky M.R., Teboul J.L., Vincent J.L. (2019). Determinants of Venous Return. Hemodynamic Monitoring. Lessons from the ICU (under the Auspices of the European Society of Intensive Care Medicine).

[3] Kellum J.A., Pinsky M.R. (1997). Rationale for Hemodynamic Monitoring. Applied Cardiovascular Physiology.

[4] Groeneveld A.B.J., Pinsky M.R. (1997). Non-Invasive Hemodynamic and Metabolic Monitoring. Applied Cardiovascular Physiology.

[5] Takala J., Pinsky M.R., Teboul J.L., Vincent J.L. (2019). Introduction to “Hemodynamic Monitoring”. Hemodynamic Monitoring. Lessons from the ICU (under the Auspices of the European Society of Intensive Care Medicine).

[6] Vincent J.-L., Rhodes A., Perel A., Martin G.S., Della Rocca G., Vallet B., Pinsky M.R., Hofer C.K., Teboul J.-L., De Boode W.-P. (2011). Clinical review: Update on hemodynamic monitoring—A consensus of 16. Crit. Care.

[7] Teboul J.-L., Saugel B., Cecconi M., De Backer D., Hofer C.K., Monnet X., Perel A., Pinsky M.R., Reuter D.A., Rhodes A. (2016). Less invasive hemodynamic monitoring in critically ill patients. Intensiv. Care Med..

[8] Vincent J.-L., Einav S., Pearse R., Jaber S., Kranke P., Overdyk F.J., Whitaker D.K., Gordo F., Dahan A., Hoeft A. (2018). Improving detection of patient deterioration in the general hospital ward environment. Eur. J. Anaesthesiol..

[9] Khanna A.K. (2018). Defending a mean arterial pressure in the intensive care unit: Are we there yet?. Ann. Intensiv. Care.

[10] Futier E., Lefrant J.-Y., Guinot P.-G., Godet T., Lorne E., Cuvillon P., Bertran S., Leone M., Pastene B., Piriou V. (2017). Effect of Individualized vs Standard Blood Pressure Management Strategies on Postoperative Organ Dysfunction Among High-Risk Patients Undergoing Major Surgery. JAMA.

[11] Leone M., Asfar P., Radermacher P., Vincent J.-L., Martin C. (2015). Optimizing mean arterial pressure in septic shock: A critical reappraisal of the literature. Crit. Care.

[12] Jozwiak M., Chambaz M., Sentenac P., Monnet X., Teboul J.-L. (2020). Assessment of tissue oxygenation to personalize mean arterial pressure target in patients with septic shock. Microvasc. Res..

[13] Hernández G., Teboul J.-L. (2016). Is the macrocirculation really dissociated from the microcirculation in septic shock?. Intensiv. Care Med..

[14] Pearse R.M., Moreno R.P., Bauer P., Pelosi P., Metnitz P., Spies C., Vallet B., Vincent J.-L., Hoeft A., Rhodes A. (2012). Mortality after surgery in Europe: A 7 day cohort study. Lancet.

[15] Michard F., Bellomo R., Taenzer A. (2018). The rise of ward monitoring: Opportunities and challenges for critical care specialists. Intensiv. Care Med..

[16] Henderson W.R., Griesdale D.E., Walley K.R., Sheel A.W.W. (2010). Clinical review: Guyton-the role of mean circulatory filling pressure and right atrial pressure in controlling cardiac output. Crit. Care.

[17] Beard D.A. (2018). Assessing the Validity and Utility of the Guyton Model of Arterial Blood Pressure Control. Hypertension.

[18] Solà J., Delgado-Gonzalo R. (2019). Handbook of Cuffless Blood Pressure Monitoring.

[19] Armentano R.L., Cabrera Fischer E.I., Cymberknop L.J. (2019). Biomechanical Modeling of the Cardiovascular System.

[20] Seagar A.D., Gibbs J.M., Davis F.M. (1984). Interpretation of venous occlusion plethysmographic measurements using a simple model. Med. Biol. Eng. Comput..

[21] Michael R. (2015). Pinsky, Functional Hemodynamic Monitoring. Crit. Care Clin..

[22] Monnet X., Marik P., Teboul J.-L. (2016). Passive leg raising for predicting fluid responsiveness: A systematic review and meta-analysis. Intensiv. Care Med..

[23] Teboul J.-L., Monnet X., Chemla D., Michard F. (2019). Arterial Pulse Pressure Variation with Mechanical Ventilation. Am. J. Respir. Crit. Care Med..

[24] Michard F., Teboul J.-L. (2000). Using heart-lung interactions to assess fluid responsiveness during mechanical ventilation. Crit. Care.

[25] Kuvin J.T., Karas R.H. (2003). Clinical Utility of Endothelial Function Testing. Circulation.

[26] Rosenberry R., Nelson M.D. (2020). Reactive hyperemia: A review of methods, mechanisms, and considerations. Am. J. Physiol. Integr. Comp. Physiol..

[27] Mayeur C., Campard S., Richard C., Teboul J.-L. (2011). Comparison of four different vascular occlusion tests for assessing reactive hyperemia using near-infrared spectroscopy. Crit. Care Med..

[28] Aya H.D., Rhodes A., Fletcher N., Grounds R.M., Cecconi M. (2015). Transient stop-flow arm arterial–venous equilibrium pressure measurement: Determination of precision of the technique. J. Clin. Monit. Comput..

[29] García M.I.M., Gil Cano A., Monrové J.C.D. (2008). Arterial pressure changes during the Valsalva maneuver to predict fluid responsiveness in spontaneously breathing patients. Intensiv. Care Med..

[30] Orbegozo D., Mongkolpun W., Stringari G., Markou N., Creteur J., Vincent J.-L., De Backer D. (2018). Skin microcirculatory reactivity assessed using a thermal challenge is decreased in patients with circulatory shock and associated with outcome. Ann. Intensiv. Care.

[31] Michard F., Chemla D., Teboul J.-L. (2015). Applicability of pulse pressure variation: How many shades of grey?. Crit. Care.

[32] Jun J.-H., Chung R.K., Baik H.J., Chung M.H., Hyeon J.-S., Lee Y.-G., Park S.-H. (2019). The tidal volume challenge improves the reliability of dynamic preload indices during robot-assisted laparoscopic surgery in the Trendelenburg position with lung-protective ventilation. BMC Anesthesiol..

[33] Monnet X., Saugel B. (2018). Could resuscitation be based on microcirculation data? We are not sure. Intensiv. Care Med..

[34] Cecconi M., De Backer D., Antonelli M., Beale R., Bakker J., Hofer C., Jaeschke R., Mebazaa A., Pinsky M.R., Teboul J.L. (2014). Consensus on circulatory shock and hemodynamic monitoring. Task force of the European Society of Intensive Care Medicine. Intensiv. Care Med..

[35] Dubin A., Henriquez E., Hernández G. (2018). Monitoring peripheral perfusion and microcirculation. Curr. Opin. Crit. Care.

[36] Ince C. (2005). The microcirculation is the motor of sepsis. Crit. Care.

[37] Mesquida J., Gruartmoner G., Espinal C. (2013). Skeletal Muscle Oxygen Saturation (StO_2_) Measured by Near-Infrared Spectroscopy in the Critically Ill Patients. BioMed Res. Int..

[38] McNulty J., Born M., Pozos R.S., Kramme R., Hoffmann K.P., Pozos R.S. (2011). Near-Infrared Spectroscopy (NIRS). Springer Handbook of Medical Technology. Springer Handbooks.

[39] Martin D.S., Levett D.Z., Bezemer R., Montgomery H.E., Grocott M.P., Caudwell Xtreme Everest Research Group (2013). The Use of Skeletal Muscle Near Infrared Spectroscopy and a Vascular Occlusion Test at High Altitude. High Alt. Med. Biol..

[40] Vos J.J., Ellermann S.F., Scheeren T.W.L. (2019). Journal of Clinical Monitoring and Computing 2017/2018 end of year summary: Monitoring—And provocation—Of the microcirculation and tissue oxygenation. J. Clin. Monit. Comput..

[41] Edul V.S.K., Ince C., Navarro N., Previgliano L., Risso-Vazquez A., Rubatto P.N., Dubin A. (2014). Dissociation between sublingual and gut microcirculation in the response to a fluid challenge in postoperative patients with abdominal sepsis. Ann. Intensiv. Care.

[42] Knotzer H., Hasibeder W.R. (2007). Microcirculatory function monitoring at the bedside—A view from the intensive care. Physiol. Meas..

[43] Bartels S.A., Bezemer R., Milstein D.M., Radder M., Lima A., Cherpanath T.G., Heger M., Karemaker J.M., Ince C. (2011). The microcirculatory response to compensated hypovolemia in a lower body negative pressure model. Microvasc. Res..

[44] Bezemer R., Karemaker J.M., Klijn E., Martin D., Mitchell K., Grocott M., Heger M., Ince C. (2009). Simultaneous multi-depth assessment of tissue oxygen saturation in thenar and forearm using near-infrared spectroscopy during a simple cardiovascular challenge. Crit. Care.

[45] Lima A., Bakker J. (2005). Noninvasive monitoring of peripheral perfusion. Intensiv. Care Med..

[46] Sun S., Hayes-Gill B.R., He D., Zhu Y., Huynh N.T., Morgan S.P. (2016). Comparison of laser Doppler and laser speckle contrast imaging using a concurrent processing system. Opt. Lasers Eng..

[47] Alnawaiseh M., Ertmer C., Seidel L., Arnemann P.H., Lahme L., Kampmeier T.-G., Rehberg S.W., Heiduschka P., Eter N., Hessler M. (2018). Feasibility of optical coherence tomography angiography to assess changes in retinal microcirculation in ovine haemorrhagic shock. Crit. Care.

[48] Guerraty M., Bhargava A., Senarathna J., Mendelson A.A., Pathak A.P. (2021). Advances in translational imaging of the microcirculation. Microcirculation.

[49] Harrois A., Duranteau J. (2013). Contrast-enhanced ultrasound: A new vision of microcirculation in the intensive care unit. Crit. Care.

[50] Steinberg I., Huland D.M., Vermesh O., Frostig H.E., Tummers W.S., Gambhir S.S. (2019). Photoacoustic clinical imaging. Photoacoustics.

[51] Wang C., Li X., Hu H., Zhang L., Huang Z., Lin M., Zhang Z., Yin Z., Huang B., Gong H. (2018). Monitoring of the central blood pressure waveform via a conformal ultrasonic device. Nat. Biomed. Eng..

[52] Patel A., Epstein F.H., Kramer C.M. (2008). Evaluation of the microcirculation: Advances in cardiac magnetic resonance perfusion imaging. J. Nucl. Cardiol..

[53] Wilkins E., Wilson L., Wickramasinghe K., Bhatnagar P., Leal J., Luengo-Fernandez R., Burns R., Rayner M., Townsend N. (2017). *European Cardiovascular Disease Statistics 2017*. European Heart Network. http://www.ehnheart.org/images/CVD-statistics-report-August-2017.pdf.

[54] Armentano R.L., Legnani W., Cymberknop L.J., Brambila F. (2017). Fractal Analysis of Cardiovascular Signals Empowering the Bioengineering Knowledge.

[55] Hermeling E., Hoeks A.P., Winkens M.H., Waltenberger J.L., Reneman R.S., Kroon A.A., Reesink K.D. (2010). Noninvasive Assessment of Arterial Stiffness Should Discriminate Between Systolic and Diastolic Pressure Ranges. Hypertension.

[56] Nottin S., Walther G., Vinet A., Dauzat M., Beck L., Messner-Pellenc P., Obert P. (2006). Reproducibility of automated pulse wave velocity measurement during exercise. Running head: Pulse wave velocity during exercise. Arch. Mal. Coeur Vaiss..

[57] Argyris A.A., Nasothimiou E., Aissopou E., Papaioannou T.G., Zhang Y., Blacher J., Safar M.E., Sfikakis P.P., Protogerou A.D. (2018). Mechanisms of pulse pressure amplification dipping pattern during sleep time: The SAFAR study. J. Am. Soc. Hypertens..

[58] Zong W., Moody G.B., Mark R.G. (1998). Effects of vasoactive drugs on the relationship between ECG-pulse wave delay time and arterial blood pressure in ICU patients. Proceedings of the Computers in Cardiology 1998. Vol. 25 (Cat. No.98CH36292).

[59] Bank A.J., Wilson R.F., Kubo S.H., Holte J.E., Dresing T.J., Wang H. (1995). Direct Effects of Smooth Muscle Relaxation and Contraction on In Vivo Human Brachial Artery Elastic Properties. Circ. Res..

[60] Lin A.C., Lowe A., Sidhu K., Harrison W., Ruygrok P., Stewart R. (2012). Evaluation of a novel sphygmomanometer, which estimates central aortic blood pressure from analysis of brachial artery suprasystolic pressure waves. J. Hypertens..

[61] Reymond P., Merenda F., Perren F., Rüfenacht D., Stergiopulos N. (2009). Validation of a one-dimensional model of the systemic arterial tree. Am. J. Physiol. Circ. Physiol..

[62] Block R.C., Yavarimanesh M., Natarajan K., Carek A., Mousavi A., Chandrasekhar A., Kim C.-S., Zhu J., Schifitto G., Mestha L.K. (2020). Conventional pulse transit times as markers of blood pressure changes in humans. Sci. Rep..

[63] Jadooei A., Zaderykhin O., Shulgin V.I. Adaptive algorithm for continuous monitoring of blood pressure using a pulse transit time. Proceedings of the 2013 IEEE XXXIII International Scientific Conference Electronics and Nanotechnology (ELNANO).

[64] Cattivelli F.S., Garudadri H. Noninvasive cuffless estimation of blood pressure from pulse arrival time and heart rate with adaptive calibration. Proceedings of the 2009 Sixth International Workshop on Wearable and Implantable Body Sensor Networks.

[65] Chandrasekhar A., Yavarimanesh M., Natarajan K., Hahn J.-O., Mukkamala R. (2020). PPG Sensor Contact Pressure Should Be Taken Into Account for Cuff-Less Blood Pressure Measurement. IEEE Trans. Biomed. Eng..

[66] Sola J., Vybornova A., Fallet S., Olivero E., De Marco B., Grossenbacher O., Ignjatovic N., Favre-Bulle M., Levinson N., Siutryk N. Are cuffless devices challenged enough? Design of a validation protocol for ambulatory blood pressure monitors at the wrist: The case of the Aktiia Bracelet. Proceedings of the 2020 42nd Annual International Conference of the IEEE Engineering in Medicine & Biology Society (EMBC).

[67] Jilek J., Stork M. (2010). Cuff width alters the amplitude envelope of wrist cuff pressure pulse waveforms. Physiol. Meas..

[68] Spitz R.W., Bell Z.W., Wong V., Viana R.B., Chatakondi R.N., Abe T., Loenneke J.P. (2019). The position of the cuff bladder has a large impact on the pressure needed for blood flow restriction. Physiol. Meas..

[69] Otsuka T., Munakata R., Kato K., Kodani E., Ibuki C., Kusama Y., Seino Y., Kawada T. (2013). Oscillometric measurement of brachial artery cross-sectional area and its relationship with cardiovascular risk factors and arterial stiffness in a middle-aged male population. Hypertens. Res..

[70] Wassertheurer S., Kropf J., Weber T., Van Der Giet M., Baulmann J., Ammer M., Hametner B., Mayer C.C., Eber B., Magometschnigg D. (2010). A new oscillometric method for pulse wave analysis: Comparison with a common tonometric method. J. Hum. Hypertens..

[71] Beutel F., Van Hoof C., Rottenberg X., Reesink K., Hermeling E. (2021). Pulse Arrival Time Segmentation Into Cardiac and Vascular Intervals–Implications for Pulse Wave Velocity and Blood Pressure Estimation. IEEE Trans. Biomed. Eng..

[72] Wang G., Atef M., Lian Y. (2018). Towards a Continuous Non-Invasive Cuffless Blood Pressure Monitoring System Using PPG: Systems and Circuits Review. IEEE Circuits Syst. Mag..

[73] Pulse Wave Analysis & Arterial Stiffness. Aortic Pulse Wave Velocity (PWVao). https://www.tensiomed.com/parameters/aortic-pulse-wave-velocity-pwvao/.

[74] Salvi P., Scalise F., Rovina M., Moretti F., Salvi L., Grillo A., Gao L., Baldi C., Faini A., Furlanis G. (2019). Noninvasive Estimation of Aortic Stiffness Through Different Approaches. Hypertension.

[75] Bogatu L.I., Turco S., Mischi M., Woerlee P., Bouwman A., Korsten E.H., Muehlsteff J. (2020). A modelling framework for assessment of arterial compliance by fusion of oscillometry and pulse wave velocity information. Comput. Methods Programs Biomed..

[76] Bresch E., Muehlsteff J., Schmitt L. (2018). Cuff-induced changes of pulse arrival time: Models and experimental results. EMBEC & NBC 2017 IFMBE Proceedings.

[77] Yan Y.S., Zhang Y.T. A model-based calibration method for noninvasive and cuffless measurement of arterial blood pressure. Proceedings of the 2006 IEEE Biomedical Circuits and Systems Conference.

[78] Bank A.J., Kaiser D.R., Rajala S., Cheng A. (1999). In Vivo Human Brachial Artery Elastic Mechanics. Circulation.

[79] Butlin M., Qasem A. (2016). Large Artery Stiffness Assessment Using SphygmoCor Technology. Pulse.

[80] Holz C., Wang E. (2017). Glabella: Continuously Sensing Blood Pressure Behavior using an Unobtrusive Wearable Device. Proceedings of the ACM on Interactive, Mobile, Wearable and Ubiquitous Technologies.

[81] Reiter H., Muehlsteff J., Sipilä A. Medical application and clinical validation for reliable and trustworthy physiological monitoring using functional textiles: Experience from the HeartCycle and MyHeart project. Proceedings of the 2011 Annual International Conference of the IEEE Engineering in Medicine and Biology Society.

[82] Carek A.M., Jung H., Inan O.T. (2019). A Reflective Photoplethysmogram Array and Channel Selection Algorithm for Weighing Scale Based Blood Pressure Measurement. IEEE Sensors J..

[83] Chan C., Zhang Y. Continuous and long-term arterial blood pressure monitoring by using h-Shirt. Proceedings of the 2008 International Conference on Information Technology and Applications in Biomedicine.

[84] Visvanathan A., Sinha A., Pal A. Estimation of blood pressure levels from reflective Photoplethysmograph using smart phones. Proceedings of the 13th IEEE International Conference on BioInformatics and BioEngineering.

[85] Solà J., Adler A., Santos A., Tusman G., Suarez-Sipmann F., Bohm S.H. (2011). Non-invasive monitoring of central blood pressure by electrical impedance tomography: First experimental evidence. Med. Biol. Eng. Comput..

[86] Wei J., Luo H., Wu S.J., Zheng P.P., Fu G., Lee K. (2018). Transdermal Optical Imaging Reveal Basal Stress via Heart Rate Variability Analysis: A Novel Methodology Comparable to Electrocardiography. Front. Psychol..

[87] Bogatu L.I., Turco S., Mischi M., Muehlsteff J., Woerlee P. (2021). An Experimental Study on the Blood Pressure Cuff as a Transducer for Oscillometric Blood Pressure Measurements. IEEE Trans. Instrum. Meas..

[88] Zhang G., Gao M., Xu D., Olivier N.B., Mukkamala R. (2011). Pulse arrival time is not an adequate surrogate for pulse transit time as a marker of blood pressure. J. Appl. Physiol..

[89] Mukkamala R., Hahn J.O., Inan O.T., Mestha L.K., Kim C.S., Töreyin H., Kyal S. (2015). Toward Ubiquitous Blood Pressure Monitoring via Pulse Transit Time: Theory and Practice. IEEE Trans Biomed Eng..

[90] Balmer J.C., Pretty C.G., Davidson S., Desaive T., Kamoi S., Pironet A., Morimont P., Janssen N., Lambermont B., Shaw G.M. (2018). Pre-ejection period, the reason why the electrocardiogram Q-wave is an unreliable indicator of pulse wave initialization. Physiol. Meas..

[91] Teng X.-F., Zhang Y.-T. (2007). Theoretical Study on the Effect of Sensor Contact Force on Pulse Transit Time. IEEE Trans. Biomed. Eng..

[92] Zulliger M.A., Kwak N.T.M.R., Tsapikouni T., Nikos Stergiopulos N. (2002). Effects of longitudinal stretch on VSM tone and distensibility of muscular conduit arteries. Am. J. Physiol. -Heart Circ. Physiol..

[93] Bogatu L., Bresch E., Muehlsteff J., Smink J., Woerlee P. Insights into oscillometry: An Experimental Study for Improvement of Cuff-Based Blood Pressure Measurement Technology. Proceedings of the 2019 41st Annual International Conference of the IEEE Engineering in Medicine and Biology Society (EMBC).

[94] Bogatu L.I., Turco S., Mischi M., Schmitt L., Woerlee P., Bresch E., Noordergraaf G.J., Paulussen I., Bouwman A., Korsten H.H. (2021). Modulation of pulse propagation and blood flow via cuff inflation—New distal insights. Sensor.

[95] Selvaraj N., Jaryal A.K., Santhosh J., Anand S., Deepak K.K. (2009). Monitoring of reactive hyperemia using photoplethysmographic pulse amplitude and transit time. J. Clin. Monit. Comput..

[96] Babbs C.F. (2012). Oscillometric measurement of systolic and diastolic blood pressures validated in a physiologic mathematical model. Biomed. Eng. Online.

[97] Drzewiecki G., Pilla J.J. (1998). Noninvasive measurement of the human brachial artery pressure-area relation in collapse and hypertension. Ann. Biomed. Eng..

[98] Drzewiecki G., Hood R., Apple H. (1994). Theory of the oscillometric maximum and the systolic and diastolic detection ratios. Ann. Biomed. Eng..

[99] Cymberknop L.J., Castillo F.G., Armentano R.L. Beat to Beat Modulation of Arterial Pulse Wave Velocity Induced by Vascular Smooth Muscle Tone. Proceedings of the 2019 41st Annual International Conference of the IEEE Engineering in Medicine and Biology Society (EMBC).

[100] Salvucci F.P., Schiavone J., Craiem D., Barra J.G. (2007). Arterial wall mechanics as a function of heart rate: Role of vascular smooth muscle. J. Phys. Conf. Ser..

[101] Roca F., Iacob M., Remy-Jouet I., Bellien J., Joannides R. (2018). Evidence for a Role of Vascular Endothelium in the Control of Arterial Wall Viscosity in Humans. Hypertension.

[102] Wurzel M., Cowper G.R., McCook J.M. (1970). Smooth muscle contraction and viscoelasticity of arterial wall. Can. J. Physiol. Pharmacol..

[103] van Knippenberg L., van Sloun R.J.G., Shulepov S., Bouwman R.A., Mischi M. (2020). An Angle-Independent Cross-Sectional Doppler Method for Flow Estimation in the Common Carotid Artery. IEEE Trans. Ultrason. Ferroelectr. Freq. Control.

[104] Brattain L.J., Telfer B.A., Dhyani M., Grajo J.R., Samir A.E. (2018). Machine learning for medical ultrasound: Status, methods, and future opportunities. Abdom. Imaging.

[105] Ottesen J.T., Olufsen M.S., Larsen J.K. (2004). Applied Mathematical Models in Human Physiology.

[106] Conrad L.I., Neve M., Nutton V., Porter R., Wear A. (1995). The Western Medical Tradition: 800 BC to AD 1800.

[107] Hatib F., Jian Z., Buddi S., Lee C., Settels J., Sibert K., Rinehart J., Cannesson M. (2018). Machine-learning Algorithm to Predict Hypotension Based on High-fidelity Arterial Pressure Waveform Analysis. Anesthesiology.

[108] Landry C., Hedge E.T., Hughson R.L., Peterson S.D., Arami A. (2020). Cuffless Blood Pressure Estimation for Activities of Daily Living. Annu. Int. Conf. IEEE Eng. Med. Biol. Soc..

[109] El-Hajj C., Kyriacou P. (2020). A review of machine learning techniques in photoplethysmography for the non-invasive cuff-less measurement of blood pressure. Biomed. Signal Process. Control.

[110] Shillan D., Sterne J.A.C., Champneys A., Gibbison B. (2019). Use of machine learning to analyse routinely collected intensive care unit data: A systematic review. Crit. Care.

[111] Moss T., Lake D.E., Calland J.F., Enfield K.B., Delos J.B., Fairchild K.D., Moorman J.R. (2016). Signatures of Subacute Potentially Catastrophic Illness in the ICU. Crit. Care Med..

[112] Sun S., Bezemer R., Long X., Muehlsteff J., Aarts R.M. (2016). Systolic blood pressure estimation using PPG and ECG during physical exercise. Physiol. Meas..

[113] Kendale S., Kulkarni P., Rosenberg A.D., Wang J. (2018). Supervised Machine-learning Predictive Analytics for Prediction of Postinduction Hypotension. Anesthesiology.

[114] Scheeren T.L., Vos J. (2019). Intraoperative hypotension and its prediction. Indian J. Anaesth..

[115] Michard F., Teboul J.L. (2019). Predictive analytics: Beyond the buzz. Ann. Intensiv. Care.

[116] Greenspan H., van Ginneken B., Summers R.M. (2016). Guest Editorial Deep Learning in Medical Imaging: Overview and Future Promise of an Exciting New Technique. IEEE Trans. Med. Imaging.

[117] Zhu B., Liu J.Z., Cauley S.F., Rosen B.R., Rosen M.S. (2018). Image reconstruction by domain-transform manifold learning. Nature.

[118] Rudin C. (2019). Stop explaining black box machine learning models for high stakes decisions and use interpretable models instead. Nat. Mach. Intell..

[119] Wax D.B., Lin H.-M., Leibowitz A.B. (2011). Invasive and Concomitant Noninvasive Intraoperative Blood Pressure Monitoring. Anesthesiology.

[120] Tomašev N., Glorot X., Rae J.W., Zielinski M., Askham H., Saraiva A., Mottram A., Meyer C., Ravuri S., Protsyuk I. (2019). A clinically applicable approach to continuous prediction of future acute kidney injury. Nature.

[121] Asfar P., Meziani F., Hamel J.-F., Grelon F., Megarbane B., Anguel N., Mira J.-P., Dequin P.-F., Gergaud S., Weiss N. (2014). High versus low blood-pressure target in patients with septic shock. N. Engl. J. Med..

[122] Hall P., Gill N. (2019). An Introduction to Machine Learning Interpretability.

[123] Fong R.C., Vedaldi A. (2017). Interpretable explanations of black boxes by meaningful perturbation. Proc. IEEE Int. Conf. Comput. Vis..

[124] Pals R.A.S., Hansen U.M., Johansen C.B., Hansen C.S., Jørgensen M.E., Fleischer J., Willaing I. (2015). Making sense of a new technology in clinical practice: A qualitative study of patient and physician perspectives. BMC Health Serv. Res..

[125] Ramsingh D., Alexander B., Cannesson M. (2012). Clinical review: Does it matter which hemodynamic monitoring system is used?. Crit. Care.

[126] Michard F. (2016). Hemodynamic monitoring in the era of digital health. Ann. Intensiv. Care.

